# Association between monocyte to high-density lipoprotein cholesterol ratio and multi-vessel coronary artery disease: a cross-sectional study

**DOI:** 10.1186/s12944-023-01897-x

**Published:** 2023-08-08

**Authors:** Jiaqi Chen, Kangxiang Wu, Wanchun Cao, Jianan Shao, Mingyuan Huang

**Affiliations:** Department of Cardiology, The Second Affiliated Hospital and Yuying Children’s Hospital of Wenzhou Medical University, Yongzhong Street, Wenzhou, 325000 Zhejiang China

**Keywords:** Multi-vessel coronary artery disease, Monocyte count, High-density lipoprotein cholesterol, Monocyte to high-density lipoprotein cholesterol ratio, Smoking

## Abstract

**Background:**

Patients with multi-vessel coronary artery disease (MV-CAD) have poorer clinical outcomes than those with single-vessel coronary artery disease (SV-CAD). Solid evidence underlines that high-density lipoprotein cholesterol (HDL-C) plays a protective role and monocyte plays a negative role in coronary artery disease (CAD). However, the monocyte to high-density lipoprotein ratio (MHR) has not been studied in relation to MV-CAD.

**Methods:**

In this study, 640 patients underwent coronary angiography, of whom 225 had severe coronary artery disease. Then divide the above two groups of patients into three groups based on the MHR tertiles, respectively. Logistic regression and subgroup analysis were carried out to estimate the association between MHR and MV-CAD. The receiver operating characteristic (ROC) curve analysis was constructed by combining classic CAD risk factors with MHR in response to MV-CAD. In addition, the mediating effect of MHR between smoking and MV-CAD in suspected CAD Patients was analyzed.

**Results:**

Among the three MHR groups, a statistically discrepant was observed in the number of patients with CAD, Severe-CAD and MV-CAD (*P*_CAD_ < 0.001; *P*_Severe-CAD_ < 0.001; *P*_MV-CAD_ = 0.001) in suspected CAD patients. Furthermore, the number of patients with MV-CAD (*P* < 0.001) was different in Severe-CAD patients among three MHR groups. Non-CAD and CAD patients showed statistically discrepant in MHR levels (*P* < 0.001), and this difference also was observed between SV-CAD and MV-CAD patients (*P* < 0.001). In the analysis of suspected CAD patients, a significantly positive relationship was found between MHR and CAD, Severe-CAD, and MV-CAD (*P* for trend < 0.001). The effect of MHR on MV-CAD was consistent across all subgroups, with no significant randomized factor-by-subgroup interaction (*P*-interaction = 0.17–0.89). ROC analysis showed that the model constructed with MHR and classic influencing factors of CAD was superior to the model constructed solely based on classic influencing factors of CAD (0.742 vs.0.682, *P* = 0.002). In the analysis of Severe-CAD patients, patients with higher MHR levels had a higher risk of MV-CAD [OR (95%CI): 2.90 (1.49, 5.62), *P* for trend = 0.002] compared to patients with lower MHR. The trends persisted after adjusting for demographic (*P* for trend = 0.004) and classic influencing factors of CAD (*P* for trend = 0.009). All subgroup factors for patients with MV-CAD had no interaction with MHR (*P*-interaction = 0.15–0.86). ROC analysis showed that the model combining MHR and classic influencing factors of CAD was superior to the one including only the classic influencing factors of CAD (0.716 vs.0.650, *P* = 0.046). Assuming that MHR played a mediating effect between smoking and MV-CAD in suspected CAD patients. The results indicated that MHR played a partial mediating effect of 0.48 (*P* < 0.001).

**Conclusion:**

A higher MHR was mainly associated with multi-vessel coronary artery disease and MHR partially mediated the association between smoking and MV-CAD.

## Introduction

Despite the decline in coronary artery disease mortality over the past decade, CAD prevalence remains high [1, 2]. With the aging population and the increase in diabetes and obesity, MV-CAD accounts for 30% to 40% of patients with CAD [3]. The prevalence of left ventricular dysfunction, comorbidities, and mortality rate of MV-CAD patients are higher than those of SV-CAD [4–6]. The negative outcomes, complexity of vessel lesions, expected results of revascularization and mortality risk should be considered to determine the type of CAD revascularization [7]. The selection of MV-CAD treatments, including coronary artery bypass grafting (CABG) or simultaneous or staged percutaneous coronary intervention (PCI), is based on the complexity of the affected vessels and the comorbidities [7–9]. Due to the high incidence of MV-CAD, the poor outcomes of MV-CAD, and the complex selection of surgical methods, biological indicators are required for the early detection of patients with MV-CAD, thus optimizing the surgical method.

Chronic inflammation is an essential hallmark of atherosclerosis [10–12]. Endothelial injury, hemodynamic damage and abnormal lipid metabolism are observed in early-stage atherosclerosis, with flow-mediated inflammatory changes playing a significant role in endothelial cells [13]. In late-stage atherosclerosis, the vessel wall is infiltrated by a large number of inflammatory cytokines and macrophages, leading to plaque rupture, bleeding, and thrombosis [14, 15]. During atherogenesis, the significant source of proinflammatory components is monocyte [16, 17]. Monocytes are recruited to the atherosclerotic plaque to fill the macrophage niche [18], which occurs at all stages of atherosclerosis [19]. Although monocytes do not reach the deep regions where foam cells reside in the plaque, it precipitates the superficial expansion of lesions from the plaque shoulder [20]. Some human and mouse model studies also reported that the number of circulating blood monocyte are closely related to the formation and development of atherosclerosis [18, 21]. On the contrary, the best-known atheroprotective function of HDL-C is mainly associated with reverse cholesterol transport [22, 23]. In addition, HDL-C favors the repair and integrity of the endothelial layer by promoting vasorelaxation and inhibits cell adhesion and pro-inflammatory substance production [24]. Moreover, HDL-C performs potent antioxidant properties, relying on different kinds of mechanisms [25]. Plasma HDL-C concentrations and CAD in general populations in general populations were also strongly inversely associated in epidemiological studies [26]. Given the proinflammatory effects of monocytes and the anti-inflammatory effects of HDL-C, MHR is regarded as a novel marker of inflammation [27, 28].

The MHR model was originally proposed from a study showing a strong association between the appreciably regression in the plaque volume and an elevation in HDL‐C, a decrease in blood monocyte count, after pravastatin therapy. In addition, ΔHDL and Δmonocyte count would not affect each other [29]. Since then, MHR as a composite index had been emerging in numerous research of various diseases [30, 31], especially coronary artery disease. In terms of severity of coronary atherosclerosis, a significantly positively association between MHR and SYNTAX scores (for evaluation complexity and severity of coronary atherosclerosis) was examined in cross-sectional studies of stable CAD patients [32], Patients with SYNTAX score ≥ 23, MHR levels were higher than those with SYNTAX score < 23 [23]. In terms of effect evaluation after PCI treatment, acute ST-segment elevation myocardial infarction (STEMI) patients without reflow shown appreciably higher MHR levels than those with reflow [33]. In terms of prognosis of coronary artery disease, stent thrombosis risk increased by 2.2 times in the higher MHR group [34]. Incidence of MACEs (major adverse cardiovascular events) increased 1.4‐fold in ACS patients with higher MHR levels [35]. Differences in the incidence of MACEs were also observed in STEMI patients [36].

However, no study has investigated the association between MHR and the complexity of vessel lesions. Therefore, this study comprehensively investigated the association between MHR and MV-CAD. Three hypotheses were proposed for this purpose: assuming that MHR was associated with MV-CAD in suspected CAD patients, assuming that MHR was associated with MV-CAD in patients with Severe-CAD, and assuming that MHR played a mediating effect between smoking and MV-CAD.

## Materials and methods

### Study population

Data of patients admitted to the department of cardiology were extracted from the electronic medical record system of the Second Affiliated Hospital and Yuying Children’s Hospital of Wenzhou Medical University. This data had been used and removed the specific information of patients in another article of our department [37]. Use of this data was approved by the Institute of Institutional Research and Ethics of the Second Affiliated Hospital of Wenzhou Medical University (ethical number: 2021-k-71–01). The study included 640 suspects of coronary artery disease. The reasons for hospitalization were typical angina-like chest pain or tightness, myocardial enzyme spectrum abnormalities, troponin abnormalities, or electrocardiogram abnormalities. The exclusion criteria were previous PCI or CABG treatment, incomplete or unavailable monocyte count and HDL-C, severe trauma, major surgery, hemorrhagic disease, septic pyemia, long-term use of lipid-lowering drugs, previous history of cerebral infarction, significant hematologic disorders, immune system diseases, immunosuppressive treatment, malignant tumor, renal insufficiency (blood creatinine ≥ 133 μmol/L), and severe liver (alanine or aspartate aminotransferase three times more than the normal upper limits).

### Coronary angiography

Patients underwent coronary angiography via the radial artery approach to evaluate the stenosis degree of left anterior descending (LAD), right coronary artery (RCA), left circumflex artery (LCx) and left main coronary artery (LM). CAD is defined as coronary artery stenosis exceeding 50%, while Severe-CAD is defined as stenosis ≥ 50% for left main disease or ≥ 70% for non–left main disease [7]. Multi-vessel lesion was defined by the involvement of the epicardial segment of more than one major artery, whereas single-vessel lesion involved only one major artery. If stenosis exceeded 50% in the LM, this was counted as an obstructive disease in multi-vessel lesions (in place of the LAD and the LCx) [4, 38].

### Laboratory measurements

Blood samples were taken from the antecubital vein and hemogram was obtained before the operation. A biochemical analyzer was used to measure complete blood lipids, blood cell counts and some biochemical indicators to calculate the MHR.

### Statistical analysis

R software (Version 4.2.2) was used for statistical analysis. In baseline characteristics, continuous data were presented as mean (SD) and categorical data as frequency (percent). The results of the three groups were compared using variance analysis or the the χ2 test for categorical variables and Kruskal–Wallis test for continuous variables. In this study, a two-tailed *P* value < 0.05 was considered statistically significant.

Logistic regression was used to evaluate the association between MHR and CAD. According to the characteristics of HDL-C and monocyte impact on CAD, the lower tertile group was a reference. The results were expressed as OR and 95% CI, and the confounding factors were selected from the demographic characteristics and classic influencing factors of CAD [39]. In adjust I, the covariates of age, gender, and BMI were adjusted. In adjust II, age, gender, BMI, smoking, diabetes, hypertension, triglyceride and low-density lipoprotein cholesterol (LDL-C) were adjusted, and differences between groups were checked by trend test.

In addition, subgroup analysis was performed based on classic CAD influencing factors (e.g., gender, age, BMI, smoking, hypertension, systolic pressure, diastolic pressure, diabetes, LDL-C, triglyceride) to evaluate differences in the influence of MHR in each subgroup.

In order to further evaluate the predictive value of MHR, the model constructed by combining MHR and classic influencing factors of CAD was compared to the model constructed solely based on classic influencing factors of CAD.

Moreover, mediating effect analysis was carried out to test whether MHR mediated the effect of smoking on MV-CAD. The following three equations were used for the analysis of mediating effect.

1 $$\mathrm{Y}=\mathrm{cX}+{\mathrm{e}}_{1}$$

2 $$\mathrm{M}=\mathrm{aX}+{\mathrm{e}}_{2}$$

3 $$\mathrm{Y}=\mathrm{ c{^\prime}}\mathrm{X}+\mathrm{bM}+{\mathrm{e}}_{3}$$

(1) investigated the effect of X on Y; (2) revealed the effect of X on M; (3) explained the association between X and Y adjusted for M as well as M and Y adjusted for X; relative residuals are e1, e2, and e3 [40].

## Results

### Subject characteristics

This study recruited 640 patients in accordance with the inclusion and exclusion criteria. Table 1 displays the baseline characteristics of the patients subgrouped by MHR tertiles, with 220 patients in the low MHR group (< 0.32), 210 patients in the medium MHR group (0.32–0.49), and 210 patients in the high MHR group (> 0.49). Patients with high MHR were mostly male and showed a higher incidence of diabetes, and smoking, and had higher levels of BMI, HbA1c, triglyceride, glutamate transaminase, cereal grass transaminase, monocyte count, neutrophil count, lymphocyte count and white blood cell count; in contrast, lower HDL-C and ApoA1, cholesterol and left ventricular ejection fractions were observed in patients with high MHR. Some baseline characteristics were similar across all MHR tertiles, including age, hypertension, systolic and diastolic blood pressure, ApoB, and LDL-C.

**Table 1:** Baseline characteristics and coronary angiography results of patients with suspected CAD

Variables	The Level of MHR			*P*-value
	< 0.32(*n* = 220)	0.32–0.49(*n* = 210)	> 0.49(*n* = 210)	
BMI (kg/ m^2^)	24.04 ± 3.09	25.27 ± 3.32	25.58 ± 3.11	< 0.001
Gender				< 0.001
Female, n (%)	150 (68.18)	90 (42.86)	45 (21.43)	
Male, n (%)	70 (31.82)	120 (57.14)	165 (78.57)	
Age (years)	64.16 ± 10.54	64.16 ± 11.16	63.59 ± 12.82	0.838
Smoking, n (%)	38 (17.27)	67 (31.90)	95 (45.24)	< 0.001
HTN, n (%)	129 (58.64)	123 (58.57)	135 (64.29)	0.386
SBP (mmHg)	138.96 ± 19.17	139.79 ± 20.14	138.33 ± 20.02	0.752
DBP (mmHg)	79.78 ± 10.89	81.67 ± 12.04	81.40 ± 12.83	0.112
Diabetes, n (%)	45 (20.45)	52 (24.76)	66 (31.43)	0.032
HbA1c (%)	22.87 ± 13.40	26.13 ± 14.58	29.45 ± 17.89	< 0.001
LVEF (%)	65.32 ± 7.52	64.18 ± 7.65	62.82 ± 8.64	0.005
Lipid indicators				
ApoA1(g/L)	1.39 ± 0.25	1.26 ± 0.17	1.12 ± 0.15	< 0.001
ApoB (g/L)	0.87 ± 0.23	0.88 ± 0.27	0.87 ± 0.24	0.853
LDL-C (mmol/L)	3.08 ± 1.05	3.42 ± 6.09	2.95 ± 1.00	0.379
HDL-C (mmol/L)	1.41 ± 0.29	1.14 ± 0.28	0.94 ± 0.20	< 0.001
TG (mmol/L)	1.46 ± 0.83	1.95 ± 1.24	1.97 ± 1.42	< 0.001
TC (mmol/L)	4.92 ± 1.19	4.66 ± 1.19	4.39 ± 1.13	< 0.001
WBC (10^9^/L)	5.64 ± 1.38	6.54 ± 1.66	8.01 ± 2.30	< 0.001
NEUT (10^9^/L)	3.54 ± 1.29	4.07 ± 1.46	5.12 ± 2.10	< 0.001
LYM (10^9^/L)	1.59 ± 0.58	1.84 ± 0.61	2.10 ± 0.86	< 0.001
MONO (10^9^/L)	0.32 ± 0.09	0.45 ± 0.10	0.63 ± 0.15	< 0.001
Cr (μmol/L)	65.14 ± 14.74	71.31 ± 18.94	73.84 ± 17.99	< 0.001
ALT (U/L)	22.87 ± 13.40	26.13 ± 14.58	29.45 ± 17.89	< 0.001
AST (U/L)	24.40 ± 8.97	25.36 ± 9.89	27.32 ± 13.80	0.021
Coronary angiography results				
CAD, n (%)	64 (29.09)	115 (54.76)	117 (55.71)	< 0.001
Severe-CAD, n (%)	37 (16.82)	92 (43.81)	96 (45.71)	< 0.001
MV-CAD, n (%)	8 (3.64)	37 (17.62)	56 (26.67)	< 0.001

*Abbreviations*: *MHR* Monocyte to high-density lipoprotein cholesterol ratio, *BMI* Body mass index, *HTN* hypertension, *SBP* Systolic pressure, *DBP* Diastolic pressure, *DM* Diabetes mellitus, *HbA1c* Glycosylated hemoglobin, *LVEF* Left ventricular ejection fractions, *ApoA1* Apolipoprotein A-I, *ApoB* Apolipoprotein B, *TC* Cholesterol, *TG* Triglycerides, *LDL-C* Low density lipoprotein cholesterol, *HDL-C* High density lipoprotein cholesterol, *Cr* Creatinine, *ALT* Glutamate transaminase, *AST* Cereal grass transaminase, *WBC* White blood cell count, *MONO* Monocyte count, *LYM* Lymphocyte count, *NEUT* Neutrophil count, *CAD* Coronary artery disease, *Severe-CAD* severe coronary artery disease, *SV-CAD* Single-vessel coronary artery disease, *MV-CAD* Multi-vessel coronary artery disease

Among the 640 patients included in the analyses, a total of 296 patients had CAD, 225 patients had Severe-CAD, 125 patients had SV-CAD, and 101 patients had MV-CAD. A significant difference in the number of CAD, Severe-CAD, SV-CAD, and MV-CAD patients was observed among the three MHR groups (*P*_CAD_ < 0.001; *P*_Severe-CAD_ < 0.001; *P*_MV-CAD_ < 0.001).

Severe-CAD patients were subgrouped by MHR tertiles, with 76 patients in the low MHR group (< 0.38), 74 patients in the medium MHR group (0.38–0.55), and 75 patients in the high MHR group (> 0.55); Table 2 displays the corresponding baseline demographic. With the exception of BMI, monocyte count, neutrophil count, ApoA1, HDL-C and white blood cell count, no significant differences were observed between tertiles.

**Table 2:** Baseline characteristics of patients with Severe-CAD

Variables	The Level of MHR			*P*-value
	< 0.38 (*n* = 76)	0.38–0.55 (*n* = 74)	> 0.55 (*n* = 75)	
BMI (kg/ m^2^)	23.98 ± 2.89	25.26 ± 3.54	25.51 ± 3.02	< 0.001
Gender				< 0.001
Female, n (%)	38 (50.00)	21 (28.38)	7 (9.33)	
Male, n (%)	38 (50.00)	53 (71.62)	68 (90.67)	
Age (years)	66.96 ± 11.34	63.76 ± 12.36	63.87 ± 13.39	0.199
Smoking, n (%)	26 (34.21)	31 (41.89)	42 (56.00)	0.024
HTN, n (%)	50 (65.79)	46 (62.16)	44 (58.67)	0.665
SBP (mmHg)	145.17 ± 20.20	140.99 ± 20.73	138.05 ± 19.09	0.092
DBP (mmHg)	82.64 ± 13.29	81.15 ± 12.33	82.09 ± 13.43	0.777
Diabetes, n (%)	14 (18.42)	27 (36.49)	29 (38.67)	0.013
HbA1c (%)	6.26 ± 1.27	6.74 ± 1.56	6.74 ± 1.55	0.071
LVEF (%)	64.22 ± 8.65	63.07 ± 8.37	62.12 ± 7.80	0.296
Lipid indicators				
ApoA1 (g/L)	1.33 ± 0.22	1.20 ± 0.14	1.08 ± 0.15	< 0.001
ApoB (g/L)	0.92 ± 0.25	0.96 ± 0.34	0.86 ± 0.23	0.091
LDL-C (mmol/L)	3.25 ± 1.05	3.25 ± 1.23	2.93 ± 1.07	0.132
HDL-C (mmol/L)	1.29 ± 0.31	1.06 ± 0.17	0.87 ± 0.18	< 0.001
TG (mmol/L)	1.59 ± 0.77	2.14 ± 1.49	1.98 ± 1.43	0.072
TC (mmol/L)	5.06 ± 1.09	4.76 ± 1.39	4.33 ± 1.08	0.001
WBC (10^9^/L)	5.62 ± 1.39	6.52 ± 1.65	8.00 ± 2.29	< 0.001
NEUT (10^9^/L)	4.08 ± 1.45	4.86 ± 1.69	5.89 ± 2.67	< 0.001
LYM (10^9^/L)	1.74 ± 0.71	1.96 ± 0.67	2.10 ± 1.10	0.056
MONO (10^9^/L)	0.37 ± 0.12	0.49 ± 0.08	0.66 ± 0.16	< 0.001
Cr (μmol/L)	71.62 ± 17.56	73.84 ± 19.30	79.31 ± 18.69	0.034
ALT (U/L)	25.62 ± 14.59	27.54 ± 13.81	32.37 ± 20.64	0.039
AST (U/L)	26.24 ± 9.52	29.00 ± 15.66	29.53 ± 16.31	0.308
Coronary angiography results				
SV-CAD, n (%)	51 (67.11)	43 (58.11)	31 (41.33)	0.005
MV-CAD, n (%)	25 (32.89)	31 (41.89)	44 (58.67)	0.005

*Abbreviations*: *MHR* Monocyte to high-density lipoprotein cholesterol ratio, *BMI* Body mass index, *HTN* Hypertension, *SBP* Systolic pressure, *DBP* Diastolic pressure, *DM* Diabetes mellitus, *HbA1c* Glycosylated hemoglobin, *LVEF* Left ventricular ejection fractions, *ApoA1* Apolipoprotein A-I, *ApoB* Apolipoprotein B, *TC* Cholesterol, *TG* Triglycerides, *LDL-C* Low density lipoprotein cholesterol, *HDL-C* High density lipoprotein cholesterol, *Cr* Creatinine, *ALT* Glutamate transaminase, *AST* Cereal grass transaminase, *WBC* White blood cell count, *MONO* Monocyte count, *LYM* Lymphocyte count, *NEUT* Neutrophil count, *CAD* Coronary artery disease, *Severe-CAD* Severe coronary artery disease, *SV-CAD* Single-vessel coronary artery disease, *MV-CAD* Multi-vessel coronary artery disease

Among the 225 patients with Severe-CAD, a total of 125 patients had SV-CAD and 100 patients had MV-CAD. A significant difference was noted in the number of MV-CAD patients among the three groups.

**Fig. 1:**
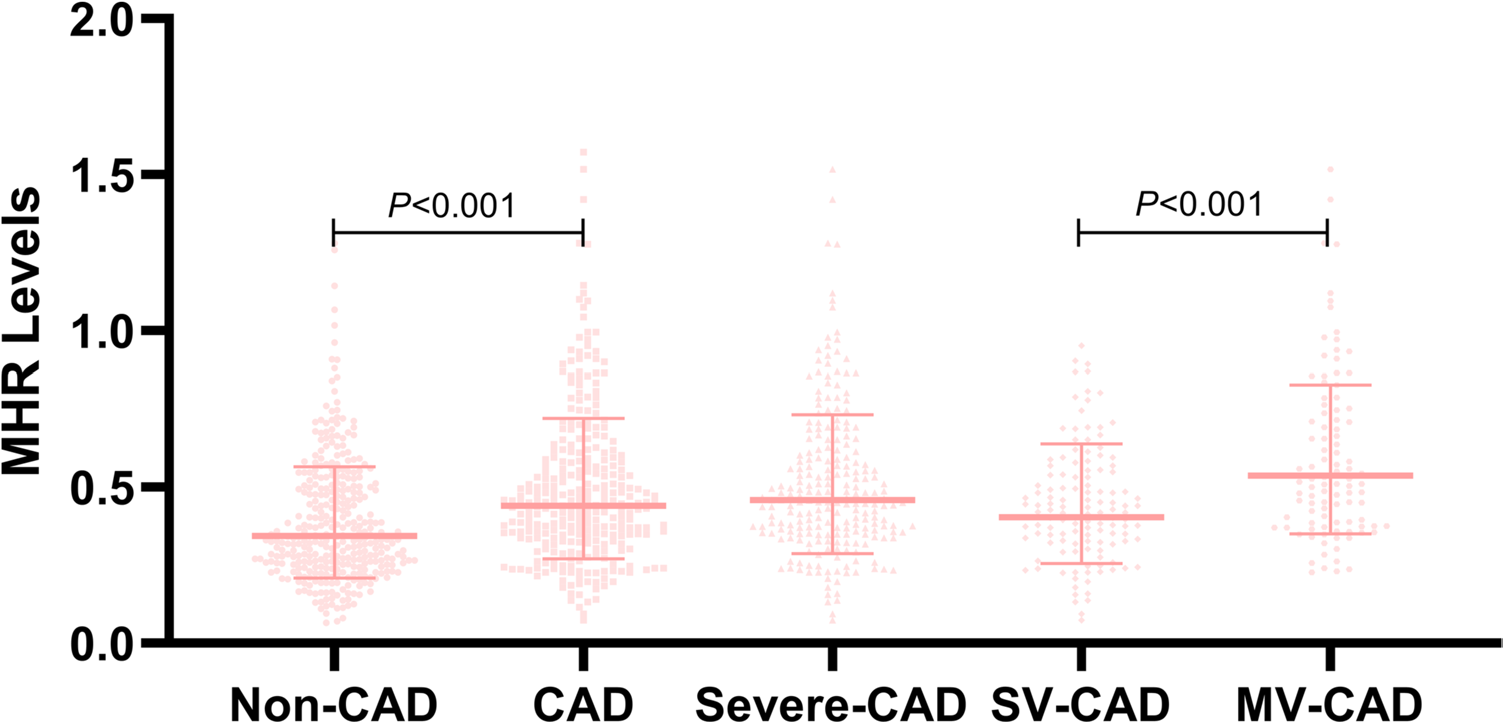
Distribution of MHR levels in patients. Abbreviations: MHR, monocyte to high-density lipoprotein cholesterol ratio; Non-CAD, non-coronary artery disease; SV-CAD, single-vessel coronary artery disease; MV-CAD, multi-vessel coronary artery disease

As shown in Fig. 1, MHR levels were presented by patients’ coronary angiography results. The MHR levels in patients with Non-CAD and CAD was significantly difference (0.39 ± 0.20, 0.50 ± 0.25, *P* < 0.001) in suspected CAD populations. In the Severe-CAD populations, the mean serum MHR levels in patients with the MV-CAD was remarkable higher than that with SV-CAD (0.44 ± 0.18, 0.59 ± 0.27, *P* < 0.001).

### Association between monocyte to HDL-C ratio and coronary angiography results in suspected CAD patients

Multivariate regression indicated a significant association between MHR and CAD, Severe-CAD, and MV-CAD in both the crude and adjusted models of suspected CAD patients, showing *P* values < 0.001. Furthermore, the number of patients with CAD was positively related to the tertiles of MHR. Compared with the control group, the OR (95% CI) values of the mid-tertile and the high tertile were 2.95 (1.98, 4.39) and 3.07 (2.06, 4.57), respectively (*P* for trend < 0.001). This upward trend remained statistically significant (adjust I: *P* for trend < 0.001; adjust II:* P* for trend < 0.001) after adjusting for confounding factors in adjust I and II. The positive association between MHR and Severe-CAD (Crude: *P* for trend < 0.001; adjust I: *P* for trend < 0.001; adjust II: *P* for trend < 0.001) and MV-CAD (Crude: *P* for trend < 0.001; adjust I: *P* for trend < 0.001; adjust II: *P* for trend < 0.001) in the suspected CAD patients also reached statistical significance (Table 3).

**Table 3:** Multivariable-adjusted association of MHR and coronary angiography results in patients who underwent coronary angiography

Variables	Crude		Adjust I		Adjust II	
	OR (95%CI)	*P* value	OR (95%CI)	*P* value	OR (95%CI)	*P* value
Association between MHR and CAD						
MHR						
Continuous	9.65 (4.44, 20.96)	< 0.001	6.36 (2.71, 14.89)	< 0.001	5.78 (2.45, 13.68)	< 0.001
Tertiles						
< 0.32	1		1		1	
0.32–0.49	2.95 (1.98, 4.39)	< 0.001	2.69 (1.77, 4.09)	< 0.001	2.72 (1.77, 4.16)	< 0.001
> 0.49	3.07 (2.06, 4.57)	< 0.001	2.45 (1.57, 3.81)	< 0.001	2.37 (1.51, 3.70)	< 0.001
*P* for trend		< 0.001		< 0.001		< 0.001
Association between MHR and Severe-CAD						
MHR						
Continuous	8.43 (3.97, 17.89)	< 0.001	5.02 (2.22, 11.36)	< 0.001	4.42 (1.94, 10.06)	< 0.001
Tertiles						
< 0.32	1		1		1	
0.32–0.49	3.86 (2.47, 6.02)	< 0.001	3.50 (2.20, 5.58)	< 0.001	3.43 (2.14, 5.49)	< 0.001
> 0.49	4.17 (2.67, 6.50)	< 0.001	3.28 (2.02, 5.32)	< 0.001	3.09 (1.89, 5.03)	< 0.001
*P* for trend		< 0.001		< 0.001		< 0.001
Association between MHR and MV-CAD						
MHR						
Continuous	21.95 (9.01, 53.43)	< 0.001	17.80 (6.78, 46.74)	< 0.001	16.18 (6.08, 43.07)	< 0.001
Tertiles						
< 0.32	1		1		1	
0.32–0.49	5.67 (2.57, 12.49)	< 0.001	5.38 (2.40, 12.05)	< 0.001	4.94 (2.20, 11.11)	< 0.001
> 0.49	9.64 (4.46, 20.80)	< 0.001	9.64 (4.46, 20.80)	< 0.001	7.47 (3.32, 16.83)	< 0.001
*P* for trend		< 0.001		< 0.001		< 0.001

Crude, no adjustment; Adjust I: age, gender, BMI, smoking, diabetes, hypertension, triglyceride and LDL-C were adjusted

*Abbreviations*: *MHR* Monocyte to high-density lipoprotein cholesterol ratio, *OR* Odds ratio, *CI* Confidence interval, *CAD* Coronary artery disease, *Severe-CAD* Severe coronary artery disease, *MV-CAD* Multi-vessel coronary artery disease

### Subgroup analysis of the association between MHR and MV-CAD in suspected CAD patients

**Fig. 2:**
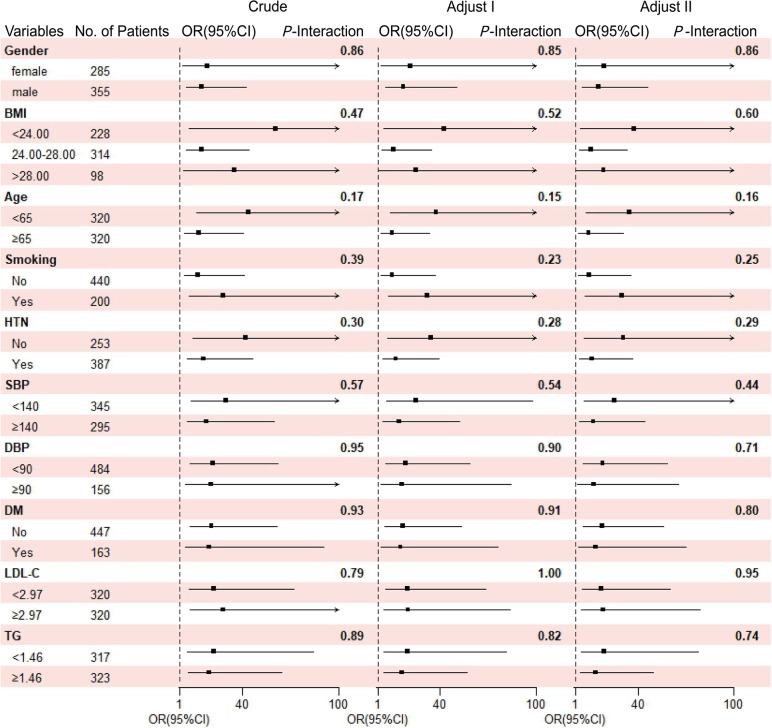
Association between MHR and MV-CAD to demographic characteristics and classic influencing factors of CAD in suspected CAD patients. Notes: Crude, no adjustment; Adjust I, each subgroup adjusted factors (age, gender and BMI) except the subgroup factors themselves; Adjust II: each subgroup adjusted factors (age, gender, BMI, smoking, hypertension, diabetes, LDL-C, and triglyceride) except the subgroup factors themselves. Abbreviations: BMI, body mass index; HTN, hypertension; SBP, systolic pressure; DBP, diastolic pressure; DM, diabetes mellitus; LDL-C, low density lipoprotein cholesterol; TG, triglycerides; OR, odds ratio; CI, confidence interval

An analysis of subgroups was performed to further evaluate the association of MHR and MV-CAD in suspected CAD patients with respect to potential confounders. All subgroup factors for patients with MV-CAD had no interaction with MHR (*P*-interaction = 0.17–0.89). After adjusting the demographic characteristics and classic influencing factors of CAD, no significant heterogeneity was observed among all subgroups (adjust I:* P*-interaction = 0.15–1.00; adjust II: *P*-interaction = 0.16–0.95) (Fig. 2).

### ROC curve analysis for monocyte to HDL-C ratio reflected MV-CAD in suspected CAD patients

**Fig. 3:**
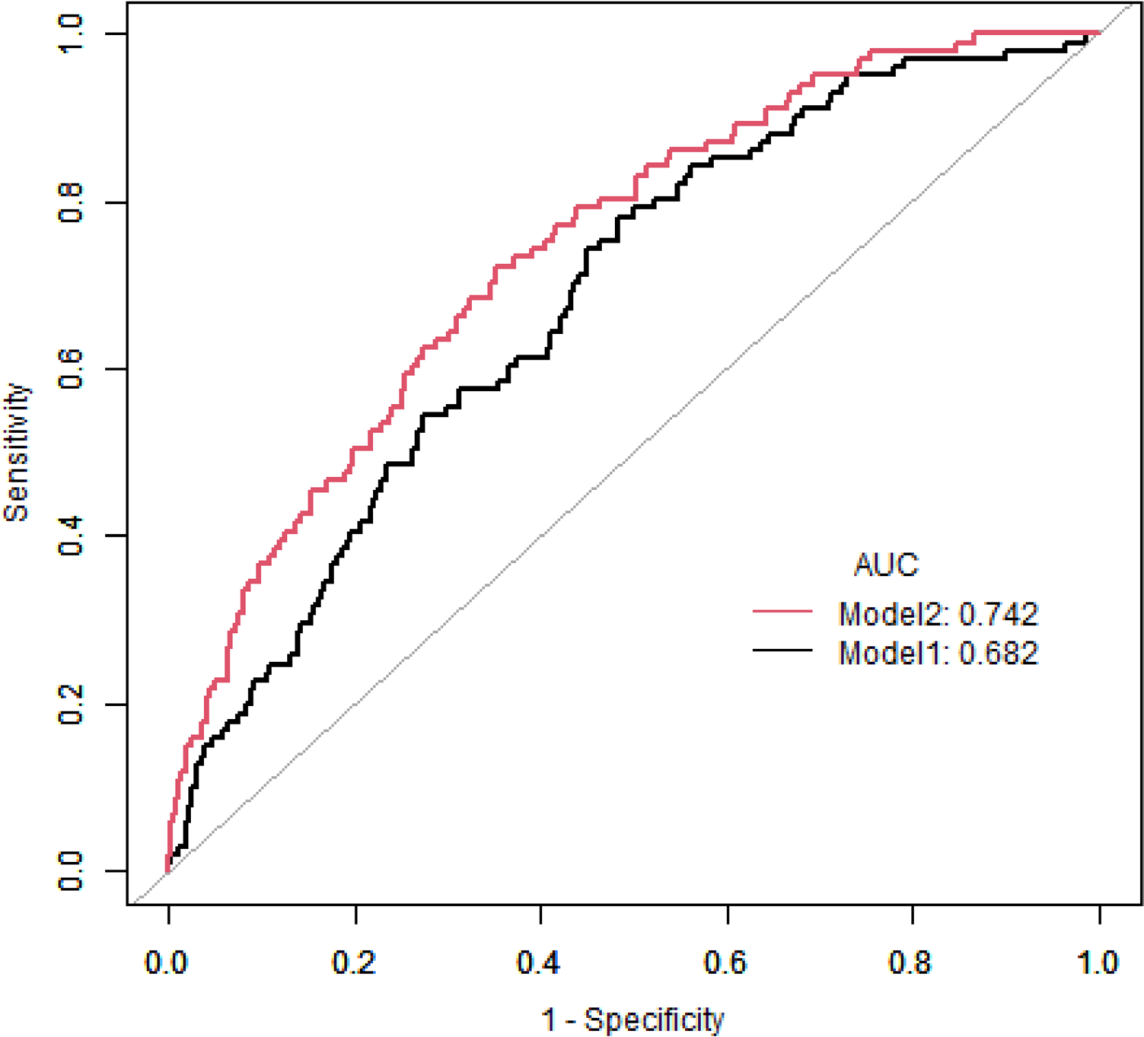
ROC curve analysis for MHR reflected MV-CAD in suspected CAD patients. Model 1: A model constructed based on age, gender, BMI, smoking, systolic pressure, diabetes, low-density lipoprotein cholesterol (LDL-C), and triglyceride. Model 2: A model constructed based on age, gender, BMI, smoking, systolic pressure, diabetes, low-density lipoprotein cholesterol (LDL-C), and triglyceride with MHR. Abbreviations: ROC: receiver operating characteristic; MHR, monocyte to high-density lipoprotein cholesterol ratio; AUC, Area Under Curve

The ROC curve analysis was carried out, and the analysis results are shown in Fig. 3. Compared to the model constructed with only classic influencing factors of CAD (age, gender, BMI, smoking, diabetes, hypertension, triglyceride and LDL-C), the model combining the classic influencing factors of CAD and MHR was superior (0.742 vs.0.682, *P* = 0.002).

### Association between monocyte to HDL-C ratio and MV-CAD in severe-CAD patients

Table 4 displays the multivariate regression analysis results for the effects of MHR on MV-CAD in Severe-CAD patients. When the MHR was a continuous variable, MHR was associated with MV-CAD whether the confounding factors were adjusted (*P* < 0.001). When the MHR was set as a nominal variable, a positive association was found between MHR and MV-CAD (*P* for trend = 0.002). This upward trend remained statistically significant (adjust I: *P* for trend = 0.004; adjust II:* P* for trend = 0.009), after adjusting the demographic characteristics and classic influencing factors of CAD.

**Table 4:** Odds ratio(95%CI) for MV-CAD by MHR in Severe-CAD patients

	Crude		Adjust I		Adjust II	
MHR	OR (95%CI)	*P* value	OR (95%CI)	*P* value	OR (95%CI)	*P* value
Continuous	20.03 (5.18, 77.45)	< 0.001	25.02 (5.68, 110.30)	< 0.001	23.70 (5.18, 108.45)	< 0.001
Tertiles						
< 0.38	1		1		1	
0.38–0.55	1.47 (0.76, 2.86)	0.256	1.44 (0.73, 2.86)	0.291	1.15 (0.56, 2.37)	0.694
> 0.55	2.90 (1.49, 5.62)	0.002	2.90 (1.42, 5.94)	0.036	2.66 (1.26, 5.60)	0.010
*P* for trend		0.002		0.004		0.009

Crude, no adjustment; Adjust I: age, gender and BMI; Adjust II: age, gender, BMI, smoking, hypertension, low-density lipoprotein cholesterol, and triglyceride

*Abbreviations*: *OR* Odds ratio, *CI* Confidence interval, *MHR* Monocyte to high-density lipoprotein cholesterol ratio, *OR* Odds ratio, *CI* Confidence interval, *MV-CAD* Multi-vessel coronary artery disease

### Subgroup analysis of the association between MHR and MV-CAD in severe-CAD patients

**Fig. 4:**
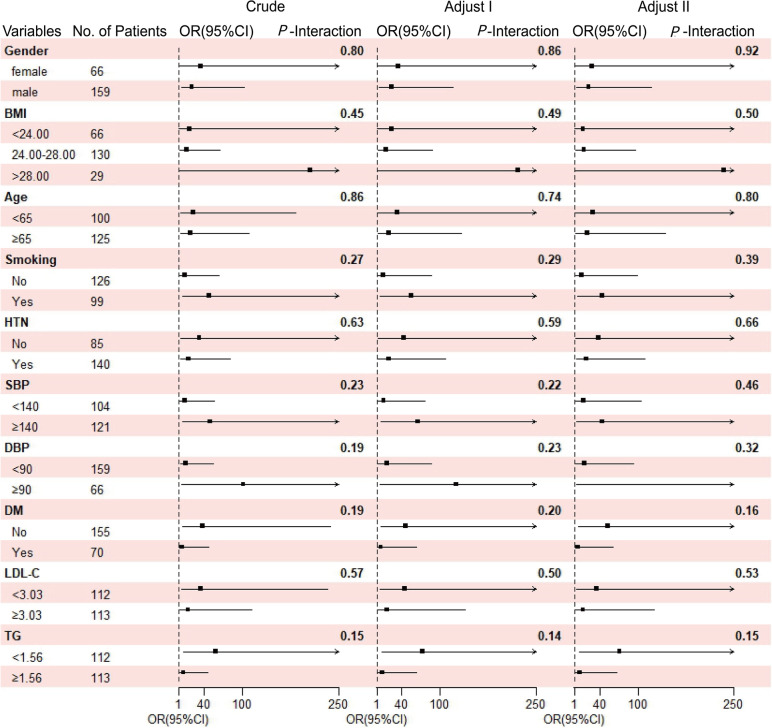
Association between MHR and MV-CAD to demographic characteristics and classic influencing factors of CAD in Severe-CAD patients. Notes: Crude, no adjustment; Adjust I, each subgroup adjusted factors (age, gender and BMI) except the subgroup factors themselves; Adjust II: each subgroup adjusted factors (age, gender, BMI, smoking, diabetes, hypertension, triglyceride and LDL-C) except the subgroup factors themselves. Abbreviations: OR, odds ratio; CI, confidence interval; BMI, body mass index; HTN, hypertension; SBP, systolic pressure; DBP, diastolic pressure; DM, diabetes mellitus; LDL-C, low density lipoprotein cholesterol; TG, triglycerides; OR, odds ratio; CI, confidence interval

Figure 4 shows the results of the stratified analyses in Severe-CAD patients in the association between MHR and MV-CAD. Regardless of adjustment for confounding factors, all subgroup factors for patients with MV-CAD had no interaction with MHR (Crude: *P*-interaction = 0.15–0.86; Adjust I: *P*-interaction = 0.14–0.74; Adjust II: *P*-interaction = 0.15–0.80).

### ROC curve analysis for monocyte to HDL-C ratio reflected MV-CAD in severe- CAD patients

**Fig. 5:**
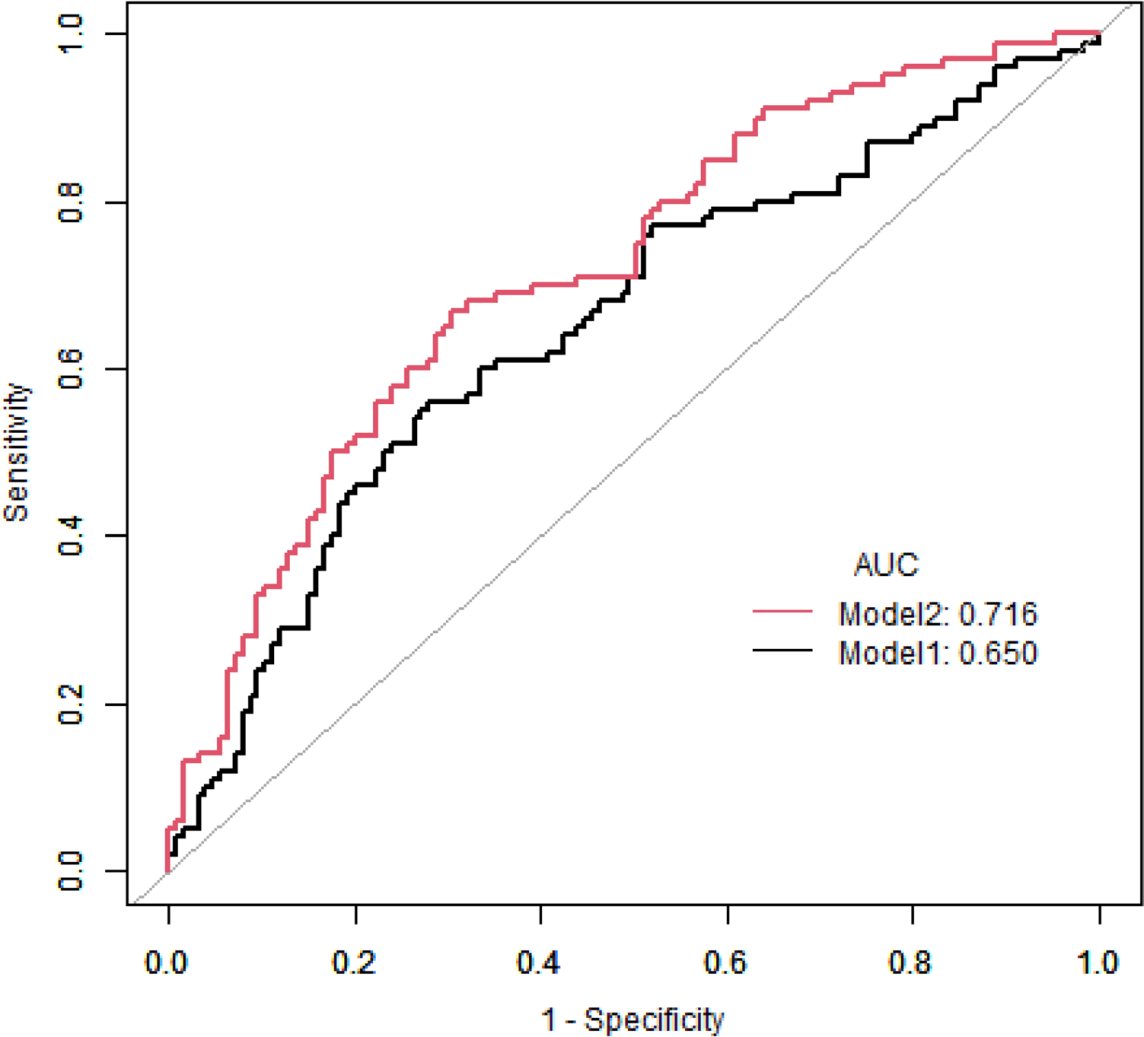
ROC curve analysis for MHR reflected MV-CAD in Severe-CAD patients. Model 1: A model constructed based on age, gender, BMI, smoking, systolic pressure, diabetes, low-density lipoprotein cholesterol (LDL-C), and triglyceride.Model 2: A model constructed based on age, gender, BMI, smoking, systolic pressure, diabetes, low-density lipoprotein cholesterol (LDL-C), and triglyceride with MHR. Abbreviations: ROC: receiver operating characteristic; MHR, monocyte to high-density lipoprotein cholesterol ratio; AUC, Area Under Curve

ROC curve analysis further to shown the potential value of MHR for CAD. The model constructed through MHR joint classic influencing factors of CAD, including age, gender, BMI, smoking, diabetes, hypertension, triglyceride and LDL-C, was superior to the model constructed solely based on classic influencing factors of CAD (0.716 vs. 0.650,* P* = 0.046) (Fig. 5).

### The mediating effect of MHR between smoking and MV-CAD in suspected CAD patients

Smoking was significantly associated with MHR in a linear regression analysis (0.12, (0.09,0.16), Table 5). In addition, smoking was found to be correlated with MV-CAD by logistic regression analysis (0.12, (0.06,0.18), Table 6).

**Table 5:** General Linear Regression on the Relationships between Smoking and MHR

	β	95% CI lower	95% CI upper	*P* value
Smoking to MHR	0.12	0.09	0.16	< 0.001

*Abbreviations*: *OR* odds ratio, *CI* confidence interval, *MHR* monocyte to high-density lipoprotein cholesterol ratio

**Table 6:** Logistic Regression on the Relationships between Smoking and MV-CAD

	OR	95% CI lower	95% CI upper	*P* value
Smoking to MV-CAD	0.12	0.06	0.18	< 0.001

*Abbreviations*: *OR* Odds ratio; *CI* Confidence interval; *MHR* Monocyte to high-density lipoprotein cholesterol ratio

MHR was found to mediate the association between smoking and MV-CAD symptoms. The average total effect was 0.11 (*P* < 0.001) and the average indirect effect was 0.05 (*P* < 0.001). However, no significant average direct effect was observed between smoking to MV-CAD symptoms in the total model (β = 0.06, *P* = 0.100). The proportion of mediating effect was 0.48 (*P* < 0.001) (Table 7).

**Table 7:** Mediating effect of MHR between smoking and MV-CAD

	Effect	95% CI lower	95% CI upper	*P* value
Total effect	0.11	0.04	0.18	< 0.001
Mediation effect	0.05	0.03	0.08	< 0.001
Direct effect	0.06	-0.01	0.12	0.100
Proportion mediated	0.48	0.27	1.16	< 0.001

*Abbreviations*: *OR* Odds ratio, *CI* Confidence interval, *MHR* Monocyte to high-density lipoprotein cholesterol ratio

## Discussion

In previous studies on CAD and MHR, including the occurrence, severity score, and prognosis [32, 33, 36], no clear association was revealed between MHR and the anatomical structure of CAD lesions. This study specifically revealed a mainly associated between MHR and MV-CAD. Analysis of suspected CAD patients, an association was observed between MHR and CAD, Severe-CAD, and MV-CAD. CAD risk was increased in patients with higher MHR, which was also observed in Severe-CAD and MV-CAD patients. Although some participants with slightly older or other risk factors were more likely to have CAD, the above association remained significant after adjusting for demographic and classic influencing factors of CAD. Moreover, there was no remarkable difference in the association between MV-CAD and MHR among subgroups. In addition, the model combining classic influencing factors of CAD and MHR showed a superior association with MV-CAD than the model constructed with only classic influencing factors of CAD. Further analysis of Severe-CAD patients, patients with SV-CAD were in the negative group. Even after adjusting for confounding factors, MV-CAD and MHR was positively associated. There was not the interaction across all subgroup factors with MHR for patients with MV-CAD. The model combining MHR and classic influencing factors of CAD was superior to the model solely including classic influencing factors of CAD. Furthermore, MHR partially mediated smoking and MV-CAD in suspected CAD patients, with a mediating effect of 0.48.

In the study, higher MHR was associated with MV-CAD in Severe-CAD patients, which was consistent with the results of a study on MHR and SYNTAX score [23]. Co-reactive MHR was related to the severity of coronary atherosclerosis. However, this study, emphasizing the association between MHR and the number of vascular lesions in CAD, provided more meaningful evidence for the treatment of CAD. Due to the complexity of treatments and poor outcomes of MV-CAD, active measures such as controlling diet, changing lifestyle habits, and avoiding infection in patients with higher MHR are of great significance in preventing the occurrence and development of CAD. In addition, timely coronary angiography, effective perioperative care, and choosing treatment methods were of great value for patients with high MHR. Monocytes are pro-inflammatory cells and play an essential role in atherogenesis. Growing evidence suggests that the monocyte count is related to the progression of atherosclerotic plaque [41, 42] and the extent of atherosclerotic entities [42, 43]. In contrast, HDL-C resists monocyte macrophages by directly counteracting their migration and removing cholesterol from macrophages to inhibit atherosclerosis [22, 23]. The close association between MHR and MV-CAD could be explained by the role of monocytes and HDL-C in atherosclerosis. However, the mid-tertile group did not differ statistically significantly from the control group in trend analysis, for which the small sample size may be responsible. Therefore, the association between MHR and MV-CAD requires further study.

Previous research revealed a close relationship between smoking and CAD, as well as an undeniable impact on blood lipids and monocytes [44]. Nevertheless, the mechanism between smoking and CAD remained controversial. Previous studies have shown that smoking is related to reduced HDL-C levels. Smoking affects lipid transport enzymes and alters HDL-C by oxidative modification. Therefore, smoking has a negative impact on both HDL-C function and quantity [45]. In addition, many studies have proven a dose-dependent response between peripheral monocytosis and smoking [46, 47]. Therefore, the mediating effect of MHR on the association between smoking and MV-CAD verified in this study. Results showed a partial mediation between smoking and MV-CAD risk by MHR.

### Strengths

When new inflammatory markers were shown to be associated with CAD, a key question was whether the marker was a close association with the treatment of the disease. Identifying the complexity of vessel lesions is crucial in CAD treatment. Most studies on MHR and CAD limited the population to participants with CAD, and the studies aimed at the prognosis of the disease. Cetin, M. S et al. found that patients with higher MHR experienced 1.4 × MACE rates than those with lower MHR in ACS patients [35]. Observations shown that the MHR could predict hospital mortality independently [36, 48]. Nevertheless, the association between MHR and the number of potentially affected coronary vessels has scarcely been studied. The study emphasized the association between MHR and the anatomical structure of CAD lesions and assessed the association between MHR and MV-CAD. The results highlighted that higher MHR was mainly associated with MV-CAD in Severe-CAD. In addition, the study focused on first-time CAD patients, minimizing confounding by pre-existing CAD conditions and related drugs. Due to the exclusion of patients with prior CAD, a very low number of patients were using cholesterol-lowering drugs.

### Limitations

However, the limitations of this study should be acknowledged. Due to the nature of this post-hoc analysis, residual confounding cannot be completely eliminated, so further research is needed. Secondly, the study only included the classic risk factors of CAD, and some novel ones were not included. Notably, the study only included some indicators of examination results. In future studies, the impact of lifestyle habits such as diet and physical activity on MV-CAD should also be excluded. Third, the use of drugs might affect the results of this study. The study excluded patients who had previously received PCI or CABG treatment, minimizing the impact of some CAD-related drugs. However, patients might be receiving long-term antiplatelet drugs for other indications, or use other cardiovascular drugs, such as antihypertensive drugs, B receptor blockers, etc. Whether these drugs impact the correlation between MHR and MV-CAD requires further verification. In addition, patient data were collected from a single site, limiting the applicability of the results to other communities, and additional research is required to verify these findings. Finally, the sample size was relatively small, resulting in underpowered analyses. A larger sample size should be included in future research to improve the accuracy of the results.

## Conclusion

Higher MHR was associated mostly with MV-CAD, whereas MHR partially mediated the association between smoking and MV-CAD.

## Data Availability

The data that support the findings of this study are available from the corresponding author.

## Abbreviations

SV-CAD: Single-vessel coronary artery disease
MV-CAD: Multi-vessel coronary artery disease
CAD: Coronary artery disease
MHR: Monocyte-to-high density lipoprotein ratio
ROC: Receiver operating characteristic
HDL-C: High density lipoprotein cholesterol
PCI: Percutaneous coronary intervention
CABG: Treatment or coronary artery bypass grafting
MACEs: Major adverse cardiovascular events
STEMI: Acute ST-segment elevation myocardial infarction
LM: Left main coronary artery
LAD: Left anterior descending
RCA: Right coronary artery
LCx: Left circumflex artery
OR: Odds ratio
CI: Confidence Interval
BMI: Body mass index
LDL-C: Low density lipoprotein cholesterol
HTN: Hypertension
SBP: Systolic pressure
DBP: Diastolic pressure
DM: Diabetes mellitus
HbA1c: Glycosylated hemoglobin
LVEF: Left ventricular ejection fractions
ApoA1: Apolipoprotein A-I
ApoB: Apolipoprotein B
TC: Cholesterol
TG: Triglycerides
Cr: Creatinine
ALT: Glutamate transaminase
AST: Cereal grass transaminase
WBC: White blood cell count
MONO: Monocyte count
LYM: Lymphocyte count
NEUT: Neutrophil count
AUC: Area Under Curve
